# The Treatment of Recent Cases of Insanity in Asylums and Private Houses

**Published:** 1887-12

**Authors:** George Fielding Blandford

**Affiliations:** London; Physician to Munster House Asylum, Etc.


### THE TREATMENT OF RECENT CASES OF INSANITY IN ASYLUMS AND PRIVATE HOUSES.*

BY GEO. FIELDING BLANDFORD, M. D., F. R. C. P., LONDON.

Physician to Munster House Asylum, Etc.

Happy is the psychologist who is not concerned with his patients until he receives them within the walls of his asylum. Many of the officers of our large public asylums are thus fortunate. It is not theirs to advise removal, to write certificates, to plan means of conveyance. They receive their patients only in hospitals fitted up with the best contrivances for the treatment and management of the insane, and can apply themselves without drawback or hindrance to the task. Very different is the life of the consultant summoned to see a patient who has been found to be insane in his own home ; to follow him from place to place, possibly by stealth, in order to obtain the necessary data to advise a plan of treatment. Although anxiously consulted by his friends, his advice is as often rejected as accepted. He is supposed to carry about with him some potent elixir which, without any restraint, will drive away the clouds and bring back the light of reason.

I do not wish to discuss here the treatment of chronic cases or cases of long standing, but only recent cases, which are, for the

*Abstract of an address read before the International Medical Congress at Washington, September, 1887. 2

most part, somewhat acute, in which there is no question as to the insanity of the patient, and in which prognosis rather than diagnosis will play the most important part. We have to consider, when called to an acute case of mania, whether it is likely to be brief or of long duration, and where and how we propose to deal with it. It behooves us to place the sufferer under such conditions that the cure may be effected as surely and as speedily as possible. In most cases a well-ordered asylum offers the best means of treatment, and where this is advisable, removal thither ought to be urged in the strongest terms. From time to time, however, we meet with patients whom, for many reasons, we may desire to save from the stigma which attaches to one who has been the inmate of an asylum. It is an objection not lightly to be put aside. Many fathers of families may be damaged in position and prospects, or, it may be, the patients are young ladies or young mothers whose future life would be affected by the reputation of having been in an asylum. The public attaches little importance to a patient being sent away into the country or kept secluded in a cottage or at the seaside, but they think a great deal of residence in an asylum. I have often seen cases of recovery from acute attacks without removal to an asylum, and when this can be achieved it is a very great advantage. I wish now to mention a few points which will enable us to determine a prognosis.

That this passing mania is recognized by psychologists is certain. Its very name, *mania transitoria*, marks its character sufficiently. Its chief characteristic is the suddenness of the attack. So marked is this that in the course of a few hours the patient may develop symptoms of a very violent or acute character. There may or may not have been premonitory symptoms; there is generally an exciting cause of some kind lighting up disorder in a brain characterized by instability and explosiveness. It frequently passes away with the same rapidity, but this is not always the case. I have known a lady pass from that condition into chronic mania, although the attack was quite sudden; and seeing that she was at once removed to an asylum, one may suppose that all that treatment could do was done. If the cause is recent, definite, and not spread over a long period of time, it is to be hoped that if judiciously treated the attack may, in a brief time, pass off. Such a cause may be a shock of some kind ; the loss of a relative, an operation, etc., sufficient to upset the equilibrium of a mind always unstable. Even where it is not possible to remove the cause, removal and seclusion will give the troubled mind time to recover its balance. Another and frequent cause of violent attacks of mania is religious excitement. Revivalist meetings are often followed by such cases. Here again we may hope that in such persons the symptoms will quickly subside.

After a hot course of spiritualism, too, I have observed outbreaks of acute mania. Two I recently met with were as violent cases of mania as one can imagine, but got well in a few weeks without removal from their homes. Another cause is protracted exercise or excessive fatigue ; also exposure to the sun for long periods of time. This was the case of an officer who had walked many hours in a hot sun and became excessively fatigued. This set up a condition of irritability which culminated in a violent quarrel with his superior officer, accompanied by delusions and a sort of mania of grandeur. When I saw him I found he had been placed in confinement as a dangerous lunatic. After going into the history of the case I thought it might not last very long, and as it turned out, my prognosis was correct. We took him away to his own home, and there he soon got well.

Very acute maniacal symptoms sometimes occur at the close or in the course of acute diseases, such as measles, variola, pneumonia, etc. They arise not when the fever is at its height, but at a later stage. It has been called the "delirium of collapse." It generally comes on on awakening from sleep. Here again the prognosis is favorable. Such an occurrence naturally causes the greatest consternation in the house, and the nurse who has been attending the patient is not always competent to deal with it. Removal from home should not be thought of until sufficient time shall have elapsed to show whether the condition is only ephemeral or is likely to prove more or less permanent. Here, too, I may mention the peculiar mania which occurs in connection with gout and rheumatism. These cases may assume an appearance of great gravity in a short time and then as suddenly disappear on an attack of the disease declaring itself. In such cases it is sometimes worth while to bring about an attack of the gout by hot fomentations to the feet or by a liberal diet.

Mania often develops as a consequence of repeated epileptic seizures. We are all familiar with the acute alcoholic delirium, *delirium tremens*. It is not uncommon and is easily recognized. It usually terminates within a week in either death or recovery. Besides this, however, we often find symptoms of insanity produced by drinking resembling those of ordinary insanity, but which passes away quickly if the drinking has not been of long standing. I had a case last year in a young woman of twenty in whom the symptoms subsided after two nights of good rest obtained by narcotics. The other was an elderly lady, stated to be addicted to taking chlorodyne, but I strongly suspect that she was in the habit of taking her chlorodyne with whisky. The fact of her drinking was kept dark, but she got well in about a fortnight, and then the whole truth came out.

The history of similar and repeated attacks will be an element of great importance in our prognosis. Some minds are essentially unstable. Just as we see so many persons who are driven into furious gusts of passion by mere trifles, so the equilibrium of some minds is upset by circumstances which under other conditions would have no significance. Of course, recovery after several attacks will not be so speedy as the first time, but it may still be hoped for with a little patience. In some people the attacks are more hypochondriacal than melancholic. Two cases have lately come under my notice ; both terminated suddenly, owing to different causes. Each tried to escape, one by running away, the other by suicide. The first succeeded in getting away to friends, where, safe from the machinations of his imaginary enemies, he soon got better. The other took poison, but only made himself very sick, and this seemed to cure him. He was packed off by the local authorities to the nearest asylum, and he wrote to me and explained the circumstances. I saw him on the following day, and he was then apparently quite well.

Now, what is there to determine the prognosis as to the duration of the attack? The temperature gives us no assistance, but the pulse may be of use. Rapid during the continuance of the excitement, when the paroxysm is over, it may fall considerably and be not much above the normal. If it does not fall, and, on the contrary, remains rapid, even when the patient is at rest, the chances are that the attack will be long and last not days, but weeks. The tongue, too, will give us help. In very violent attacks it will often remain moist and clean, but if it becomes dry, furred, and brown, with that peculiar coat which is characteristic—not of gastric disorder, but of nervous trouble—then we can hardly hope that it will pass off in two or three days. There is often a good deal in these attacks of mania of what, for want of a better name, we may call "hysterical." They are characterized by noise and violent behavior rather than by fixed ideas or delusions. If they begin suddenly, and if between the attacks the patient is rational, then we may hope, but if the violence increases and the intervals are marked by gradually forming delusion, then the look-out is bad.

Sleeplessness is always a chief symptom, and it often happens that after a long sleep, produced by a narcotic, the patient awakes recovered or in such a state as to cause no further anxiety. If the maniacal attacks do not subside in a few days or a week, and it appears probable that it will be an affair of weeks or months, what course are we to take? The friends may be opposed to our sending the patient to an asylum. The question, I have found, resolves itself often into one of cost. There are many patients noisy and troublesome who are not homicidal and who can be nursed through the attack of mania if the friends will bear the expense. In nine cases out of ten it is necessary to remove them from their immediate surroundings, so that special rooms have to be taken for the purpose elsewhere. To harness a patient to the bed by his wrists and ankles for weeks together is not treatment. There must be facilities for out-door exercise and an active and intelligent surveillance. All this means expense; but if money be no object, then it is worth trying to avoid the stigma of an asylum and the feelings incidental to detention therein. Many of these cases ultimately drift into the asylums. The friends get tired of providing the cost and trouble of looking after the patient.

Certain patients ought to be sent to the asylum at once to get the benefit of the example. Such a one will refuse to eat when kept alone, but if put with twenty others he will eat because they do, especially if there are signs that nobody cares very much whether he eats or not. When we have cases of mania of long standing and incurable it is better for the friends to send them at once to asylums; otherwise they may exhaust their means and make it worse for the patient subsequently. There is no class for which asylums are so necessary as the general paralytics. They want more attention and space than can be given at home, and the happy nature of their delusions prevents their chafiing at their confinement or feeling their position. I will now consider the treatment of depressed and melancholic patients. Here the question of suicidal tendency comes up; but it must be borne in mind that residence in an asylum is not an absolute safeguard against suicide. Nothing save vigilant and active surveillance will insure this, and that can be done as well outside as inside an asylum. Some patients will only attempt suicide if they find a tempting opportunity, while others will do it whether other people are looking on or not. Clearly some of these people can only be treated in an asylum, while the less acute can be kept in a suitable house and by skill and care nursed back to health. It is idle to attempt treatment in home associations. There is a special reason for treating some melancholic persons in an asylum. It is to re-act against that intense egotism which marks the malady, and to prevent the patient being the focus of attention. In an asylum he is only one of a crowd. In a private house the feeling of self-importance is fostered and maintained.

The cure of a patient's insanity without sending him to an asylum is satisfactory both to the friends and to the medical attendant. It is akin to the surgeon's feeling when he has succeeded in curing a diseased joint without amputation, but I am not one of those who believe that all cases of insanity can be treated in private dwellings. We all know the importance of early and suitable treatment, and often this can only be carried out in an asylum. If we do not send patients thither, it must be because we consider that they can be as well treated outside. If no improvement takes place the patient must be sent to an asylum without delay.

There is one other point, and that is the question as to how far we are justified in treating patients outside an asylum without certificates. I am glad to be spared all reference to this subject. I am told that the law in this country varies according to the State. What I think the law ought to do is to enable us to treat a case outside an asylum without certificates just as an ordinary case of *delirium tremens*, or mania, or fever. In Scotland that is allowed, but not in England, nor is it likely to be. I have thought fit to bring this subject before you, because it has often been said that lunacy doctors have no other means of treating insanity except in asylums. I cannot hope to have assisted you much within the limits of this paper in the diagnosis of such cases, but I may claim to have directed your attention to them.

